# Pedestrian Augmented Reality Navigator

**DOI:** 10.3390/s23041816

**Published:** 2023-02-06

**Authors:** Tanmaya Mahapatra, Nikolaos Tsiamitros, Anton Moritz Rohr, Kailashnath K, Georgios Pipelidis

**Affiliations:** 1 Department of Computer Science and Information Systems, Birla Institute of Technology and Science, Pilani 333031, India; 2 Ariadne Maps GmbH, Munich, Brecherspitzstraße 8, 81541 Munich, Germany; 3 Institute for Informatics, Technical University of Munich, Boltzmannstraße 3, 85748 Garching, Germany

**Keywords:** augmented reality, augmented reality navigator, Kalman filter, Global Navigation Satellite Systems, ARKit, trilateration, beacons, data fusion

## Abstract

Navigation is often regarded as one of the most-exciting use cases for Augmented Reality (AR). Current AR Head-Mounted Displays (HMDs) are rather bulky and cumbersome to use and, therefore, do not offer a satisfactory user experience for the mass market yet. However, the latest-generation smartphones offer AR capabilities out of the box, with sometimes even pre-installed apps. Apple’s framework ARKit is available on iOS devices, free to use for developers. Android similarly features a counterpart, ARCore. Both systems work well for small spatially confined applications, but lack global positional awareness. This is a direct result of one limitation in current mobile technology. Global Navigation Satellite Systems (GNSSs) are relatively inaccurate and often cannot work indoors due to the restriction of the signal to penetrate through solid objects, such as walls. In this paper, we present the Pedestrian Augmented Reality Navigator (PAReNt) iOS app as a solution to this problem. The app implements a data fusion technique to increase accuracy in global positioning and showcases AR navigation as one use case for the improved data. ARKit provides data about the smartphone’s motion, which is fused with GNSS data and a Bluetooth indoor positioning system via a Kalman Filter (KF). Four different KFs with different underlying models have been implemented and independently evaluated to find the best filter. The evaluation measures the app’s accuracy against a ground truth under controlled circumstances. Two main testing methods were introduced and applied to determine which KF works best. Depending on the evaluation method, this novel approach improved the accuracy by 57% (when GPS and AR were used) or 32% (when Bluetooth and AR were used) over the raw sensor data.

## 1. Introduction

The idea of Augmented Reality (AR) can be summarised in the simplest way as augmenting the real-world with computer-generated information. This poses an interesting base problem for all systems that attempt to provide an AR experience. To augment the real-world, it must be perceived, at least in some ways. As technological innovation progresses, Augmented Reality (AR) becomes an increasingly exciting topic. What used to be a futuristic field of research is slowly getting to the mass market. HMDs for AR are a heavily researched field, but not yet ready for the average consumer. Smartphones, on the other hand, are prevalent, and current-generation devices offer video see-through AR capabilities. Even though a smartphone AR experience feels less natural, it showcases the potential of AR and can be seen as a precursor to HMD AR. Both Apple and Google have recently published frameworks for their mobile operating systems that enable AR apps. They are called ARKit and ARCore and offer roughly the same features. Apple ships every sold phone with an app called Measure, which is powered by ARKit and can measure the size of real-world objects. Smartphones perceive the real-world by precisely tracking the pose (position and orientation) in space and detecting elements of the environment with the camera. This enables digital content to be attached to the real-world. Tracking the smartphone’s motion is vital so digital content stays attached to its real-world position. Local positioning works reasonably well for small confined areas, but there is currently no capability to connect the local position precisely to a global position. The only option for obtaining a global position is GPS, which is only accurate to a certain degree (about 5 m).

In this paper, we contribute by attempting to solve the problem of inaccurate global positioning by fusing local motion information with global positioning data. ARKit gathers motion information via the camera and the Inertial Measurement Unit (IMU). The global positioning data come from GPS and a custom Bluetooth-based positioning system. The latter is necessary for indoor scenarios where GPS does not work.

We describe the PAReNt ios app, which showcases pedestrian navigation via AR. Navigation is based on global coordinates and, therefore, a well-suited application. The results of this work enable not just this example, but many other AR applications are possible, which could improve the quality of life. In principle, digital information can be attached to coordinates and displayed at a certain position. Recommender systems based on location and viewing angle would be apt examples for this. The outside wall of a museum could display a preview of what’s inside. If precise positioning is connected with Internet Of Things (IoT) systems, smart decisions can be made by gadgets, such as turning off the light when the user leaves the room.

### Outline

The rest of the manuscript is structured in the following way. Section 2 explores related work in the fields of AR navigation, GNSS, and Bluetooth-based positioning approaches. Section 3 explains the background knowledge that this work is based on and the details of the data fusion method, the Kalman Filter (KF). Section 4 explains how the described problem of precise positioning was approached. Section 5 describes the important implementation details. Section 6 discusses the evaluation and the quality of the results. Section 7 discusses the limitations of this work and how they can be improved in the near future, and Section 8 concludes the manuscript.

## 2. Related Work

### 2.1. Augmented Reality Navigation

The idea of navigation as an application of augmented reality systems has existed for a while. The potential advantages of AR navigation in military aircraft has already been investigated [1]. It was concluded that information about basic navigation, flight information, and possible targets could be superimposed in the helmet-mounted sights of pilots. A wearable augmented reality system [2] has already been presented that was location-aware and could annotate the user’s view with geographical information. The system consisted of multiple separate sensors and computing devices that are portable, but rather cumbersome to use. The goal was to develop a system that displays digital location or object-based information indoors and outdoors while giving the user the possibility to enhance and extend this information. One part of the application was a user guidance system that displayed a path in the user’s view.

An AR navigation system for cars and a modified version for pedestrian navigation have already been realised [3]. The system consists of a Personal Digital Assistant (PDA), which acts as a video see-through display, a camera as the visual input, a GPS sensor, an orientation tracker, a navigational device, and a computational unit (laptop). In the car setup, the camera is mounted behind the front mirror, and the display is close to the dashboard. The camera is placed directly behind the PDA in the pedestrian handheld setup. The computational unit collects all sensor data, the video stream, and the path data from the navigational device. After putting them together, the visual navigation data are shown on the PDA display in the form of semitransparent lane markings on the camera image.

More recent projects are using the growing capabilities of smartphones [4]. SunMap+ is a prototype available on the Android operating system for indoor AR navigation. The application uses a custom-created 3D map of university buildings and facilitates the Vuforia software development kit (SDK). The landmark recognition capabilities of the latter are used to determine the users’ position. Additionally, a Pedestrian Dead Reckoning (PDR) system is used when the Vofuria SDK fails. The PDR system uses the accelerometer to detect step patterns and the magnetometer to obtain the direction of movement. This system, like all IMU-based systems, is prone to accumulating errors, often called drift. Singh et al. [5] proposed an AR-based navigation system that uses a smartphone. The spatial data are stored as a CSV file, and the location identification is performed using a GPS receiver and the stored data. A route calculation is made when the destination is entered by the user, and the route is displayed on the user’s screen. The location of the user is obtained solely based on the GPS data, and this can be prone to error. Multiple data inputs for finding out the location can be used to provide more accurate navigation.

### 2.2. GNSS Improvements

Even though the Global Navigation Satellite System (GNSS) is accurate enough for many problems, higher accuracy and precision are always desired. The integration of the Inertial Measurement Unit (IMU) with GNSS using the Kalman filter has been discussed in the literature for better accuracy [6,7]. A body-worn system has been presented, which uses vision in addition to GPS and a low-cost IMU [8]. A FAST feature detector is used in combination with the BaySAC algorithm to match and compute a homography matrix of the camera movement. The computed information is then integrated with the data captured by the IMU. It was shown that the vision and IMU combination resulted in an approximately 1m error after 60 s and less than a 3m error after 6 min. It was concluded that this improves the standalone IMU navigation radically, but uses much computing power.

As smartphones evolved, they had GPS, IMU, and more computing power. Researchers have combined GNSS and IMU in smartphones [9]. Emphasis has been placed on situations with partial GNSS availability. The focus lies in comparing different kinds of Kalman filters and testing them in simulated and real scenarios. They developed and implemented four Kalman-based methods. The first is a classic Kalman Smoother (KS), which incorporates position, heading, and step count with a fixed step length. The second Kalman smoother with a variable Step Length (KS SL) extends the first with a heuristic to incorporate the variable step length. The third and fourth are nonlinear Kalman filter extensions, namely the Extended Kalman Filter (EKS) and the Unscented Kalman Filter (UKS). The latter two incorporate the step length naturally and do not need an additional heuristic. With full GPS coverage, none of the filters stand out with significantly better results than the others. It could be argued that the UKS has a slight advantage. With only partial GPS coverage, it becomes clear that the inclusion of a variable step length makes a difference because the error of KS is about double that of the others. However, even when using a variable step length, none of the remaining filters stand out significantly. The walk was conducted by starting indoors, leaving the building, walking outdoors, turning around, and re-entering the building. The generally high error levels were regarded by the authors due to high GPS errors in the transition phases from outdoor to indoor [9].

### 2.3. Indoor Positioning Using Bluetooth

Using wireless networks to determine indoor position has been greatly researched [10]. Researchers have developed RADAR, one of the earliest systems based on WaveLan, the predecessor of WiFi [10]. All the existing approaches in positioning via wireless sensor networks have been summarised [11]. The focus lies on concepts independent of the wireless technology used. One of the concepts is trilateration. Bluetooth-based indoor location approaches have been evaluated [12]. They use a Received Signal Strength Indicator (RSSI) trilateration approach. To convert the RSSI to distance in meters, they tested their Bluetooth devices. Several measurements at different distances were made and combined with the radio propagation model. With the results of the trilateration, they deployed a gradient filter and were able to reduce their average error from 5.87 m to 2.67 m. Since this approach often takes average values over certain periods of time, it is not very suited for moving targets.

Over time and with new Bluetooth standards, more systems have been developed [13]. A system that combined Bluetooth Low-Energy (BLE) and dead reckoning for positioning via a smartphone has also been evaluated. Low-cost BLE beacons were used, and as a first step, their RSSI behaviour was examined. It was seen that the RSSI can vary greatly, and filtering is needed to deal with that. The path loss model was then used to turn the filtered values into distances in meters. For positioning, the signals of three or more beacons were fed into a multilateration model that uses the least-squares method to minimise residuals. The PDR was developed based on an accelerometer step detection that registers peaks in acceleration with a fixed step length of 74 cm. The magnetometer was used to detect the direction of walking of the user. As the last step, the results from the trilateration were combined with the results of the dead reckoning with a KF. The researchers evaluated the developed system in an office corridor. Eight beacons were deployed, and the path had a length of about 30 m.

## 3. Background

### 3.1. ARKit

ARKit [14] is a framework developed and maintained by Apple on the iOS platform that enables users to develop AR-based applications using the front and back cameras. This work only uses the back camera, for which ARKit provides features such as tracking of the environment, a coordinate system to register objects, horizontal surface detection, and more. The world tracking capabilities are powered by visual–inertial odometry [15], which combines information from the IMU and information gathered by analysing the camera video stream. The latter is performed by extracting features from each video frame and tracking their positions across frames. Altogether, it results in the capability to compute how the camera and, therefore, the whole smartphone moves in space across time. This means, as long as ARKit runs, the position (in meters) and orientation (as a vector) of the device are available, but only in comparison to the start position and orientation. It was realised in the following way: As soon as ARKit starts, it puts the origin of a local coordinate system at the position in space where the camera is located at that moment. In the local coordinate system, the x-axis points to the right and left, the y-axis points up and down, and the z-axis points out the front of the device, all relative to the position of the camera when ARkit starts. The position and orientation of the coordinate system do not change automatically during an ARKit session, but can be changed by the app developer.

### 3.2. Bluetooth Beacons

Bluetooth beacons are small devices that broadcast an identifier using the Bluetooth protocol. The broadcasting is implemented via Bluetooth low-energy proximity sensing and is independent of the receiver. The identifier of a beacon is programmable and consists of a Universally Unique IDentifier (UUID), a major and a minor value. The latter two are 16 bits each. The distance of a beacon to the Bluetooth signal receiver is usually approximated by using the RSSI in combination with a path loss model.

### 3.3. Trilateration

**Figure 1:**
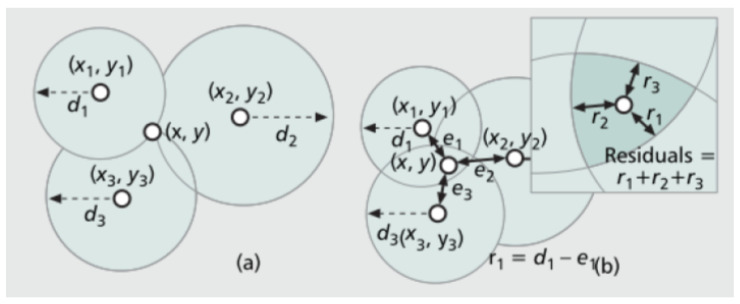
Schematic example of trilateration as in [11]. (**a**) illustrates the normal trilateration process of calculating a node's position with the help of the intersection of three circles. (**b**) illustrates the real-world scenario where the circles do not intersect precisely at one position but at many positions due to inaccurate position information, which makes estimating a node's position very difficult.

Trilateration is the process of determining the location of a point based on distances to three known locations. When the distances are not known precisely, the problem can be as visualised in Figure 1. In the real-world, measurements are imprecise and prone to noise up to a certain level. Reference [13] tackled this problem in the following way. The difference between the real radius $r_{i}$ and the measured radius $m_{i}$ can be set as $v_{i}$ = $r_{i}$ − $m_{i}$. The location x,y is the result that assumes a minimal squared measuring error, mathematically expressed as

$$\left(x,y\right)=min(\sum^{i}\left(v_{i}^{2}\right))$$

### 3.4. GNSS

Global Navigation Satellite Systems (GNSSs) are satellite systems that send radio signalsto the Earth, which can be used for location and navigation purposes. There are different levels of accuracy for the different satellite systems and different methods of computing the location, but in general, an accuracy of about 5 m is presumed. The received data are provided via a framework called *CoreLocation*.

### 3.5. Kalman Filter

This is a well-known algorithm to produce estimates of unknown variables. An optimal Kalman gain functionality that adapts the gain on the fly, based on the current uncertainty values, as well as the noise of prediction and measurement, is used. The equation below describes the prediction step.

$${\hat{x}}_{k|k-1}=F_{k}{\hat{x}}_{k-1|k-1}+B_{k}u_{k}$$

Here, ${\hat{x}}_{k-1|k-1}$ is the the state estimate at time k−1 given all observations at times before and including k−1. $F_{k}$ is the state transition model at time *k*. $B_{k}$ is the control model, and $u_{k}$ is the control vector. ${\hat{x}}_{k|k-1}$ is the newly predicted state.

$$P_{k|k-1}=F_{k}P_{k-1|k-1}F_{k}^{T}+Q_{k}$$

This equation changes the error covariance matrix in line with the predictions of state estimates. $P_{k-1|k-1}$ is the error at k−1 with all information available till k−1. $F_{k}$ is the state transition model. $Q_{k}$ is the covariance process of the noise.

The update step is broken down into five different equations, which result again in an updated state estimation and covariance matrix.

$${\tilde{y}}_{k}=z_{k}-H_{k}{\hat{x}}_{k|k-1}$$

The above equation contains $z_{k}$, the measurement at time *k*. $H_{k}$, the observation model, transforms a state into the observation space. $x_{k|\hat{K}-1}$ is the state estimate from the prediction step.

$$S_{k}=H_{k}P_{k|k-1}H_{k}^{T}+R_{k}$$

The next equation takes the predicted error covariance matrix $P_{k|k-1}$ and transforms it with the observation model ($H_{k}$) from the left and the transposed observation model ($H_{k}^{T}$) from the right. In addition to the measurement noise $R_{k}$, this results in $S_{k}$ being able to be described as the error covariance in the observation space, which includes the uncertainty of the measurement.

$$K_{k}=P_{k|k-1}H_{k}^{T}S_{k}^{-1}$$

This equation computes the optimal Kalman gain, by comparing two error covariance matrices.

$${\hat{x}}_{k|k}={\hat{x}}_{k|k-1}+K_{k}{\tilde{y}}_{k}$$

This equation uses the gain to update the state estimate. $y_{k}$ describes the difference between prediction and measurement in observation space.

$$P_{k|k}=\left(I-K_{k}H_{k}\right)P_{k|k-1}$$

This equation updates the error covariance based on information from the Kalman gain. If the gain is close to zero, $I-K_{k}H_{k}$ is close to the Identity (*I*), resulting in an error covariance close to the prediction covariance error. A high Kalman gain close to one can only occur when the measurement uncertainty is very low, and this then results in a lower error covariance than the predicted error covariance.

## 4. Conceptual Approach

**Figure 2:**
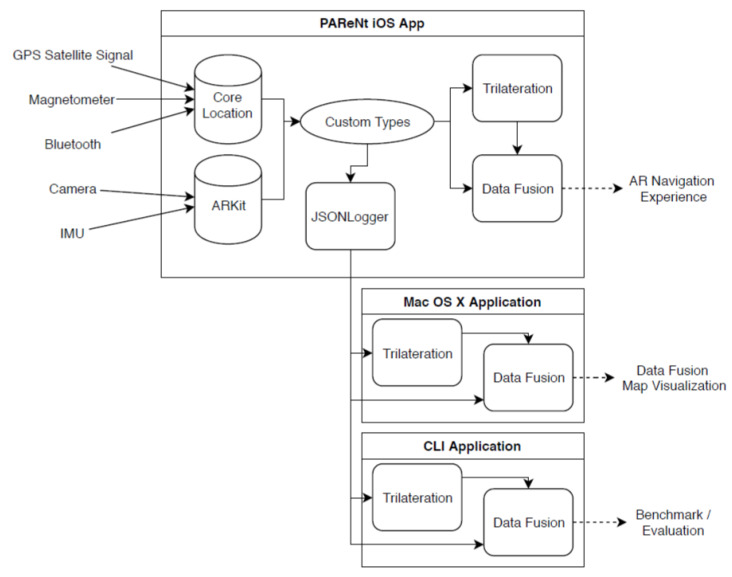
Overview of the approach.

This section gives an overview of how the proposed problem of improved positioning data for AR navigation is approached (Figure 2).

The PAReNt iOS app captures sensor data, converts them to custom types, saves them via JSON, and performs the trilateration and data fusion. The saved data are used by the Mac OS X and CLI applications.

### 4.1. Bluetooth Beacon Trilateration

Battery-powered short-range beacons sold by the company Avvel were used. To convert from the RSSI to distances, a simplified model was used, which approximates the signal propagation model with a second-order polynomial.

### 4.2. Data Fusion

**Figure 3:**
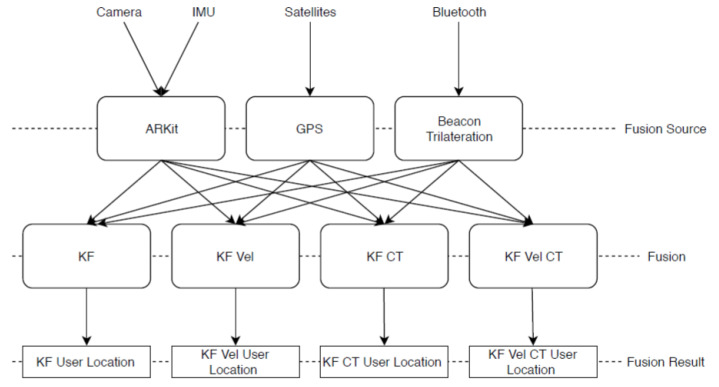
Overview of data fusion.

A general overview of how the different sensor data are fused together can be found in Figure 3. These raw data come in from the camera, the IMU, satellites, and via Bluetooth. These data are then preprocessed in ARKit, CoreLocation, and the beacon trilateration module. Four independent filters all take in the fusion source data and produce their own results.

#### 4.2.1. Kalman Filter 1

In the prediction phase, it was assumed that the state stays the same and there is no control. The covariance of the process noise is determined by one parameter *n*, which is set to meters. $n_{lat}$ and $n_{lng}$ compose the parameter *n*, converted in latitude–longitude space. The conversion between latitude, longitude, and meters is different depending on where the user is located in the world. The equation below summarises all definitions for the prediction phase in this KF.

$$\hat{x}=\left[\begin{array}{c} lat\\ lng \end{array}\right]\;F=\left[\begin{array}{cc} 1 & 0\\ 0 & 1 \end{array}\right]\;B=0_{2,2}\;u=0_{2,1}\;Q=\left[\begin{array}{cc} n_{lat} & 0\\ 0 & n_{lng} \end{array}\right]$$

When there is new ARKit sensor data available, the measurement ($z_{k}$) is composed of $\Delta a_{k,lat}$ and $\Delta a_{k,lng}$. The displacement from the last $\Delta a_{lat,k-1}$ in meters is taken and converted back into latitude space to compute a new latitude value. The same is performed for the longitude values. By doing this, the observation model ($\underset{-}{H}$) is very simple, just the identity matrix. The measurement noise ($R_{k}$) is determined by one parameter mA in meters, which is then converted to the coordinate space. The equation below summarises the definitions for the update phase:

$$z_{k}=\left[\begin{array}{c} \Delta ar_{k,lat}\\ \Delta ar_{k,lng} \end{array}\right]\;H=\left[\begin{array}{cc} 1 & 0\\ 0 & 1 \end{array}\right]\;R=\left[\begin{array}{cc} mA_{\mathrm{lat}\;} & 0\\ 0 & mA_{\mathrm{lng}} \end{array}\right]$$

For new GPS data, the modelling is similar, but no Δ value needs to be taken for the measurement. Therefore, $gps_{k,lat}$ just describes the latitude GPS sensor data at time *k*. The measurement noise mG can change with time. CoreLocation provides information about the accuracy of each measurement, depending on the GPS signal strength and more. The equation below summarises the definitions required for the GPS update step:

$$z_{k}=\left[\begin{array}{c} gps_{k,lat}\\ gps_{k,lng} \end{array}\right]\;H=\left[\begin{array}{cc} 1 & 0\\ 0 & 1 \end{array}\right]\;R_{k}=\left[\begin{array}{cc} mG_{k,lat} & 0\\ 0 & mG_{k,lng} \end{array}\right]$$

#### 4.2.2. Kalman Filter 2: KF Vel

This model includes the velocities in the latitude and longitude direction. The function used describes the distance between two steps ($\Delta s_{k}$), as the time between the steps ($\Delta t_{k}$) times the last velocity ($v_{k}-1$). The state ($\hat{x}$) is now four-dimensional, because of the additional velocity variables. The state transition model ($F_{k}$) implements this velocity function in the latitude and longitude direction and is now different in each step *k* because of $\Delta t_{k}$. As in the simple KF filter, no control method (B,u) is used.

$$\hat{x}=\left[\begin{array}{c} \;\mathrm{lat}\;\\ \mathrm{lng}\\ v\;\mathrm{Lat}\;\\ vLng \end{array}\right]\;F_{k}=\left[\begin{array}{cccc} 1 & 0 & \Delta t_{k} & 0\\ 0 & 1 & 0 & \Delta t_{k}\\ 0 & 0 & 1 & 0\\ 0 & 0 & 0 & 1 \end{array}\right]\;B=0_{4,4}\;u=0_{4,1}$$

The process noise was assumed to be happening because of a constant acceleration $\underset{-}{a}$ in the latitude and longitude direction, based on Newton’s laws of motion $\Delta s_{k}=\frac{1}{2}a\Delta t^{2}+v_{k-1}$ and $v_{k}=a\Delta t+v_{k-1}$. Therefore, in matrix form, all together, this becomes $x_{k}=F_{k}x_{k-1}+G_{k,lat}a_{lat}+G_{k,lng}a_{lng}$ with $G_{k,lat}$ and $G_{k,lng}$ defined as:

$$G_{k,lat}=\left[\begin{array}{c} \frac{1}{2}\Delta t_{k}^{2}\\ 0\\ \Delta t_{k}\\ 0 \end{array}\right]\;G_{k,\mathrm{lng}}=\left[\begin{array}{c} 0\\ \frac{1}{2}\Delta t_{k}^{2}\\ 0\\ \Delta t_{k} \end{array}\right]$$

This results in the following process noise covariance matrix:

$$Q_{k}=G_{k,lat}G_{k,lat}^{T}\sigma_{lat\;}^{2}+G_{k,lng}G_{k,lat}^{T}\sigma_{\mathrm{lng}}^{2}=\left[\begin{array}{cccc} \frac{1}{4}\Delta t_{k}^{4}\sigma_{\mathrm{lat}\;}^{2} & 0 & \frac{1}{2}\Delta t_{k}^{3}\sigma_{\mathrm{lat}\;}^{2} & 0\\ 0 & \frac{1}{4}\Delta t_{k}^{4}\sigma_{lng}^{2} & 0 & \frac{1}{2}\Delta t_{k}^{3}\sigma_{\mathrm{lng}\;}^{2}\\ \frac{1}{2}\Delta t_{k}^{3}\sigma_{\mathrm{lat}\;}^{2} & 0 & \Delta t_{k}^{2}\sigma_{\mathrm{lat}\;}^{2} & 0\\ 0 & \frac{1}{2}\Delta t_{k}^{3}\sigma_{\mathrm{lng}}^{2} & 0 & \Delta t_{k}^{2}\sigma_{\mathrm{lng}}^{2} \end{array}\right]$$

where σ is the standard deviation of *a* and $G_{k,lat}a_{lat}+G_{k,lng}a_{lng}\;N\left(0,Q_{k}\right)$. For ARKit and the GPS data, $z_{k}$ and $R_{k}$ are constructed the same way as the last filter. The definitions are:

$$\begin{array}{cc} & z_{k}=\left[\begin{array}{c} \Delta ar_{k,lat}\\ \Delta ar_{k,lng} \end{array}\right]\;H=\left[\begin{array}{cccc} 1 & 0 & 0 & 0\\ 0 & 1 & 0 & 0 \end{array}\right]\;R_{k}=\left[\begin{array}{cc} mA_{lat} & 0\\ 0 & mA_{lng} \end{array}\right]\\ & z_{k}=\left[\begin{array}{c} gps_{k,lat}\\ gps_{k,lng} \end{array}\right]\;H=\left[\begin{array}{cccc} 1 & 0 & 0 & 0\\ 0 & 1 & 0 & 0 \end{array}\right]\;R=\left[\begin{array}{cc} mG_{k,lat} & 0\\ 0 & mG_{k,lng} \end{array}\right] \end{array}$$

#### 4.2.3. Kalman Filter 3: KF CT

The main difference between the first two filters and this one is that they use the ARKit data as the control input, instead of the measurement input. The KF CT has a two-dimensional state consisting just of latitude and longitude values. The control vector $\Delta ar_{k}$ is the displacement (difference) from the last ARKit value in meters. This is converted into the latitude=-longitude space. The control model is an identity matrix since it only connects the latitude and longitude values.

$$\hat{x}=\left[\begin{array}{c} lat\\ lng \end{array}\right]\;F=\left[\begin{array}{cc} 1 & 0\\ 0 & 1 \end{array}\right]\;B_{k}=\left[\begin{array}{cc} 1 & 0\\ 0 & 1 \end{array}\right]\;u_{k}=\left[\begin{array}{c} \Delta ar_{k,lat}\\ \Delta ar_{k,lng} \end{array}\right]\;Q=\left[\begin{array}{cc} n_{lat\;} & 0\\ 0 & n_{lng} \end{array}\right]$$

The update step only consists of one GPS part, because the ARKit data are already used in the prediction step. This results in exactly the same definitions as for the KF GPS update step.

#### 4.2.4. Kalman Filter: Four KF Vel CT

The KF Vel CT filter is a mixture between KF Vel and KF CT, and it uses a four-dimensional state similar to KF Vel, but uses the ARKit data as the control vector. The state $\hat{x}$, the state transition model $F_{k}$, and the error covariance of the process noise (Qk) are defined as in KF Vel. The difference is in the control model and the control vector. The AR data are used in the control vector to adjust a change in velocity between the last step (k−1) and the current (k). The velocity during the step *k* is computed by $\Delta var_{k}=\frac{\Delta ar_{k}}{\Delta t_{k}}$, where $\Delta ar_{k}$ is the difference in metersdetected by ARKit between the last step and the current step. $\Delta var_{k}$ shows the change in velocity. The control model propagates that changed velocity into the state estimation velocity and adjusts the position as well. The control model and control vector are defined as:

$$B_{k}=\left[\begin{array}{cccc} 0 & 0 & \Delta t_{k} & 0\\ 0 & 0 & 0 & \Delta t_{k}\\ 0 & 0 & 1 & 0\\ 0 & 0 & 0 & 1 \end{array}\right]\;u=\left[\begin{array}{c} 0\\ 0\\ \dot{\Delta }{\mathrm{var}}_{k,lat}\\ \dot{\Delta var},\;\mathrm{lng}\; \end{array}\right]$$

The update step is similar to KF CT, only considering the GPS data, because the ARKit data were already handled in the update step. The definitions are identical to the KF Vel definitions for the GPS update.

### 4.3. Single Instruction Multiple Data

The Kalman filter is based mainly on matrix operations such as multiplications and inversions. This work used an approach to speed up the computations by computing matrix operations in parallel. This was performed by using Single-Instruction Multiple-Data (SIMD) types. ARM Neon, which is part of the CPU in the iPhone, was used for this purpose.

### 4.4. Augmented Reality Navigation

The navigation part was approached by displaying path markers on the ground. The route information in the form of global latitude–longitude coordinates was obtained with Apple’s MapKit framework. After the user chooses a destination, MapKit provides a pedestrian route, which is converted into a path. The path is displayed in the user’s augmented reality view, based on the user’s current location and orientation, which are based on the results of the Kalman filters. The path is visually pinned to the ground by setting the z-axis coordinate (height) to the same value as the ground. This information about the ground is obtained by using ARKit’s horizontal plane detection feature.

## 5. Implementation

### 5.1. Overview

**Figure 4:**
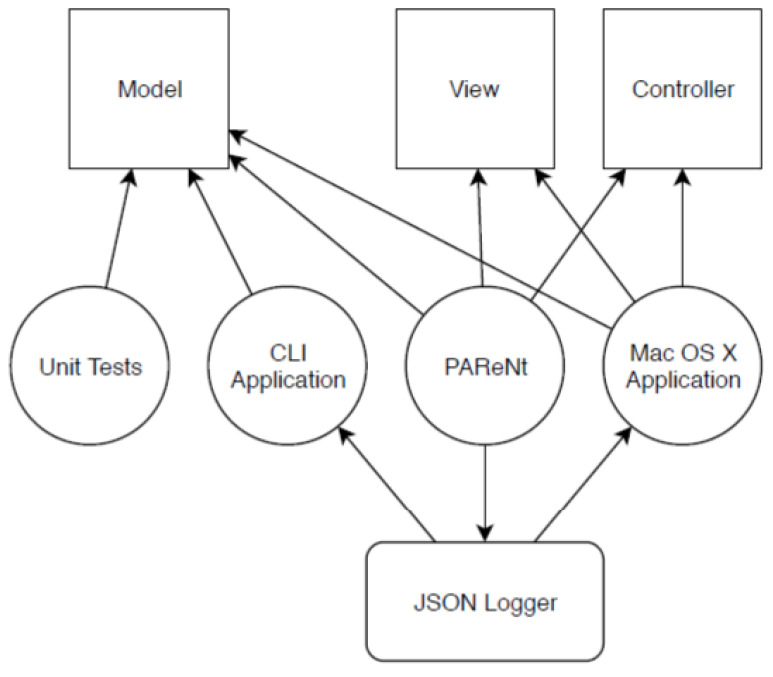
Overview of the implementation.

The MVC pattern was used to decouple functionalities and, therefore, keep the codebase easily extendible (Figure 4). The connection between the three modules was realised in Apple’s implementation of the observer pattern, named NotificationCenter. When the model computes something new, it notifies all observers. The view was set up as a model observer and, therefore, was able to render the newly computed information.

#### 5.1.1. iOS Application Parent

The main iOS app is called PAReNt. This app uses almost all parts of the later described code, besides some specific views of the Mac application and some evaluation methods of the CLI application.

#### 5.1.2. Mac OS X GUI Application

This application is a native Mac OS X application that acts as a visualisation and debugging tool. Via the JSONLogger, the collected sensor data from the iOS app are fed into this tool. This was valuable during development because the KFs could be tested on Mac OS X without actually performing a new walk again.

#### 5.1.3. CLI Application

The CLI application is a benchmarking tool to measure the performance of the KFs. It has two main tasks: the first is to use a batch of collected walks and evaluate all primary sensor inputs, as well as the KFs. The second task is optimising the various parameters of the four KFs.

### 5.2. Model

**Figure 5:**
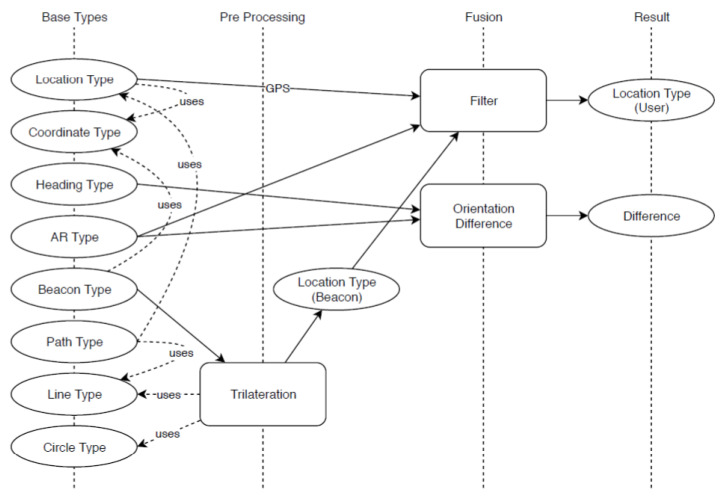
Overview of the model.

The model as part of the MVC is shared by all the applications, and its internal architecture is shown in Figure 5. The base types are defined to provide a custom implementation instead of using Apple’s internal types (resulting in tighter coupling to Apple’s ecosystem). The second stage is the pre-processing and consists only of the trilateration, takes the Bluetooth beacon data and computes a location. The next step is the fusion, which includes all four KFs.

#### 5.2.1. Location Type

The location type represents a certain location on the Earth’s surface (latitude–longitude, altitude, accuracy of values). Furthermore, this type encapsulates the functionality needed to convert between geographic coordinates and X, Y, and Z displacement coordinates. The explicit handling of the latitude and longitude values, as well as all algorithms needed for geometry on a model of the Earth are implemented in the coordinate type, which is the only other base type that the location type is using.

#### 5.2.2. Coordinate Type

The coordinate type carries the latitude and longitude values and provides algorithms for dealing with the geometry on the Earth’s surface. There are several models to describe the shape of the Earth. The following three models were implemented:

Equirectangular projection: This model projects the Earth’s surface on a rectangular plane. This model has several problems, which result in straight lines on the globe not translating into straight lines on the projection. The same is true for distances.

Earth as a sphere: Modelling the Earth as a sphere results in an increase in accuracy, especially for larger distances. Only the radius is needed to describe all properties of the sphere. The radius is often approximated as 6,378,137 m. The geometric algorithms are based on [16] and are therefore computationally more complex.

Earth as an ellipsoid: The Earth’s shape can be approximated better by using an ellipsoid instead of a sphere.

The geometric algorithms were based on [17], which are two iterative methods. The methods give a higher accuracy, but are computationally even more complex than the algorithms on a sphere.

#### 5.2.3. Heading Type

The heading type represents the orientation on a map. This is an angle between 0 and 360 degrees. The type is directly initialised by the main controller with data coming from CoreLocation. The data come from the internal magnetometer sensor, which measures the Earth’s magnetic field.

#### 5.2.4. Augmented Reality Type

This type is a custom representation of the information that is provided by Apple’s ARKit framework. The framework computes additional information based on the camera and the inertial measurement unit. This AR type combines information about the camera position and orientation in reference to a local coordinate system. Additionally, this type computes a property called xzOrientation upon initialisation. It is an angle that represents the phone’s general-looking direction on the Earth’s surface.

#### 5.2.5. Beacon Type

The beacon type represents all data that are received from the Bluetooth beacons. For every beacon, there is an identifier and the RSSI. The location of each beacon is supplied via a key-value store, which assigns a coordinate to each beacon identifier. The actual distance from the sensing device to a beacon is derived from the signal strength. This highly depends on which beacons are used and how strong their signal is. The beacons that were used were calibrated by measuring the signal strength at a close distance, a 1 m and 6 m distance. The RSSI was 40 for a close distance, 60 for 1 m, and 90 for 6 m. To convert between the RSSI (r) and distance (d), a second-order polynomial of the form $d=ar^{2}+br+c$ was used, which resulted in the parameters a=7,b=11, and c=36.

#### 5.2.6. Path Type

The path type holds navigation data about the route a user has to take to get to the destination. This is realised by a list of locations that embody checkpoints that the user is passing. The path type was also used to have the ground truth during the evaluation.

#### 5.2.7. Line Type

This type represents a line between two coordinates. It is directly used in trilateration and provides methods to intersect two lines.

#### 5.2.8. Circle Type

**Figure 6:**
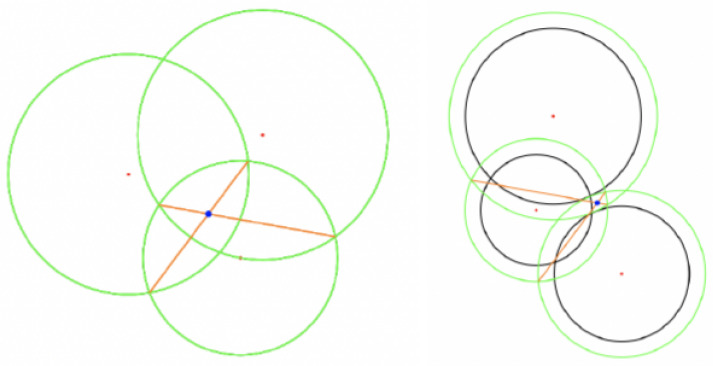
Overview: Beacon type dependencies.

This work used the properties of intersecting circles. If two circles are intersecting twice, then a line between the two intersection points can be drawn. If there are three circles that each intersect twice with each other, the resulting lines of the intersection points are intersecting themselves at a point (Figure 6).

Beacon location module: The result from the trilateration gives a specific user location, but no accuracy value. The beacon location module adds an accuracy value by taking the sum of the difference of the found solution to the three distance circles. Therefore, if something went wrong in the trilateration, this value will be very high, indicating a bad accuracy. It is best understood as: accurate to *x* meters. This module then broadcasts the trilateration solution with the accuracy value to everyone who is listening.

#### 5.2.9. Kalman Filters

**Figure 7:**
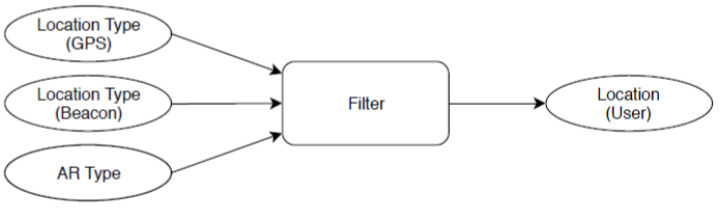
Overview: Kalman filter data flow.

Figure 7 shows a simple overview of how each filter takes in GPS, beacon, and AR data and fuses them together into one user location. The specific filters were derived from one generic filter class, which implements the prediction and update step. The generic class uses a custom matrix type internally. The matrix type implements all operations needed, such as multiplication, inversion, transposing, and more.

#### 5.2.10. Orientation Difference

**Figure 8:**
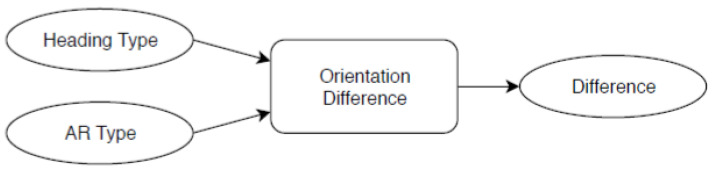
Overview: orientation difference data flow.

The orientation difference module is a KF that holds a current orientation difference value (Figure 8). It takes two inputs, the magnetometer (heading type) and the xzOrientation of the AR type, then calculates the difference. If the difference is greater than 0, this means the local coordinate system of ARKit got out of sync with the magnetometer data. The KF was used here to smooth the difference.

### 5.3. View

#### 5.3.1. Map View

**Figure 9:**
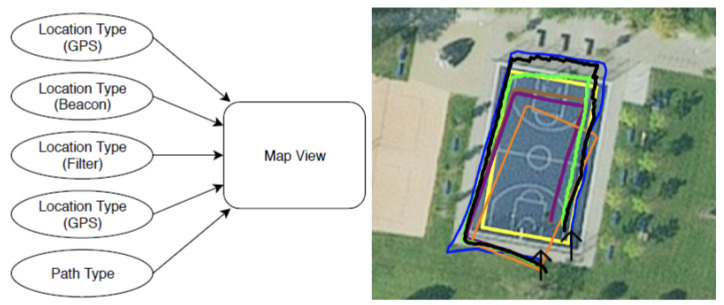
Overview of the map view data flow (**left**); example map view (**right**).

This view shows the results of all four KFs on a satellite image of the current location. The raw GPS, beacon AR data, and path are also visualised. In Figure 9, the right side shows an example of what that map view looks like. This view is the main view of the Mac application and the lower part of the debugging version of PAReNt.

#### 5.3.2. AR View

**Figure 10:**
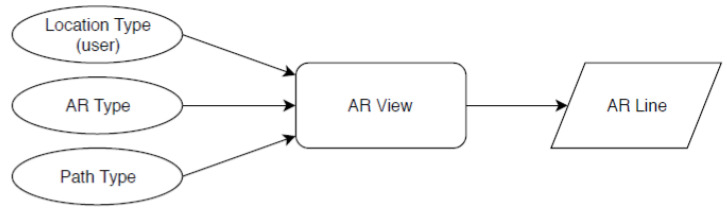
Overview AR View data flow.

This view is used in the iOS app to display the path in the camera image depending on the user’s location (Figure 10). It is basically the live image that the back-facing camera captures, augmented with a blue path. It is realised via ARKit internal functionalities and a custom type called ARLine.

### 5.4. Controller

The controller as part of MVC serves as a direct interface to the operating system. The following controllers described are only used by the iOS application and use functionalities specific to the platform.

#### 5.4.1. Main Controller

The main controller is the main interface between the underlying operating system and the application. It is the starting point of the application and sets up the JSON Logger, Kalman filter, and beacon trilateration module. The main controller configures and manages the iOS framework CoreLocation. Once the framework is started, the main controller captures all incoming data and processes them in the following ways:

Location data: The incoming location data are first converted from the Apple type CLLocation to the custom location type. Then, the data are distributed via the mechanisms of the observer pattern to every module that is interested.

Magnetometer data: Apple intends Bluetooth beacons to be a positioning mechanism where GPS is not available. Therefore, beacons are also managed via CoreLocation. CoreLocation collects all beacon data and provides them to the application once a second. The beacon type is then initialised with these data.

#### 5.4.2. AR Controller

**Figure 11:**
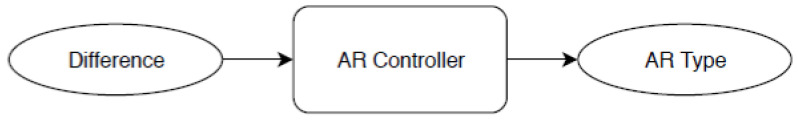
Overview of the AR controller data flow.

The AR controller serves as an interface to ARKit. It initialises the AR session and registers the event handlers for incoming data sensed by ARKit. As soon as ARKit processes a new frame, the resulting data are converted into an AR type and distributed to everyone that is listening to view the observer pattern (Figure 11). The coordinate system is also rotated here if the orientation difference module detects an inconsistency. It also directs the AR view. If a new path is broadcast (by the navigation controller), the path coordinates are converted into the AR space.

#### 5.4.3. Navigation Controller

The navigation controller’s main task is to provide the search bar for searching and choosing a navigation destination. When a navigation destination is chosen, it finds the corresponding route, converts it into a path type, and broadcasts it.

### 5.5. JSON Logger

**Figure 12:**
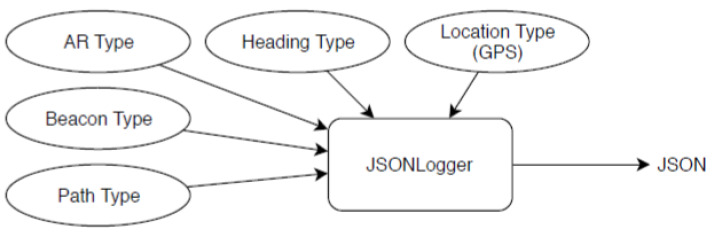
Overview of the JSONLogger data flow.

The JSONLogger listens to all the raw sensor events, as well as custom system messages and logs them (Figure 12). The sensor events are location (GPS), magnetometer, AR-type data, and beacons. The system message includes the start and end of evaluation runs and path-related data. The events are encoded into JSON objects and are then saved in the phone’s persistent memory.

### 5.6. Unit Tests

Several critical functionalities of this project cannot be solved analytically. The unit tests described in this section are mainly used to make sure correct results are produced. With the results of these tests, a medium level of accuracy was chosen. The high accuracy option did not yield any significant accuracy benefit at smaller distances (below 1 km), but resulted in a measurable performance decrease. The low-accuracy option, on the other hand, decreased in accuracy with growing distances, but did not increase performance significantly.

## 6. Evaluation

The evaluation of our work is described in this section. The two datasets and the testing methods that were used are described in the first section. The next four sections describe the evaluations of different types of input data and the performance of the proposed KFs.

### 6.1. Datasets and Testing Methods

Both datasets contain ground truths and sensor data captured from walks. During the walk, the phone was held in front of the chest with a slight tilt forward. We tried to walk at a steady pace without stopping in between with as little deviation from the path as possible. After the coordinates were taken from the satellite images, the distances between them were calculated and compared with real-world measurements to ensure that the satellite image was not distorted.

#### 6.1.1. Dataset and AR

**Figure 13:**
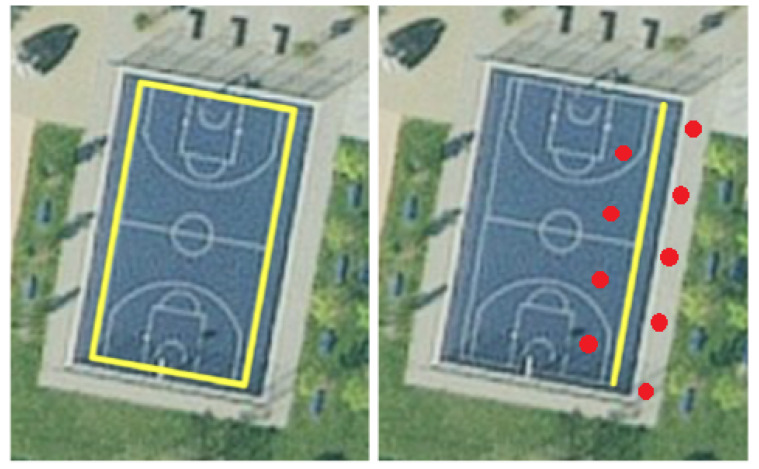
Ground truth/path of Dataset 1 (**left**) and Dataset 2 (**right**).

This is recorded data from walks around a basketball court. During recording, no beacons were deployed; hence, the recording consisted only of GPS and augmented reality data. The ground truth was the outer boundary of the court. Each dataset entry was the captured data of one walk starting from the southeast corner, around the court, in a clockwise direction. Figure 13 (left) shows the basketball court with the ground truth marked in yellow.

#### 6.1.2. Dataset 2: Beacons, GPS, and AR

This dataset was created only along one sideline of the court. Additionally, nine beacons were deployed on the right and left sides of this path. The distance between each beacon and the path was 3 m, and the distance between the beacons on each side was 6 m. Finally, the left side had a 3 m offset to the right side, resulting in a zigzag pattern. This can be seen in Figure 13 with the path in yellow and the beacons in white.

#### 6.1.3. Testing Method 1: Distance to Ground Truth

This method measures the distance of each recorded location to the ground truth. For example, if the first GPS location is one meter next to the start point of the ground truth, this results in an error of 1 m. For example (Table 1), it can be seen that the recorded GPS signal deviates on average 1.74 m (averaged over all walks of Dataset 1). To assess the performance of the GPS and the Kalman filter, the Mean-Squared Error (MSE) in the last column of the mentioned table was used. There is one downside to this method. If a filter produces results that are just staying at the start location and do not move at all, this method would result in a very low error, even though this filter performed poorly.

#### 6.1.4. Testing Method 2: Distance to Correct Point on Ground Truth

**Figure 14:**
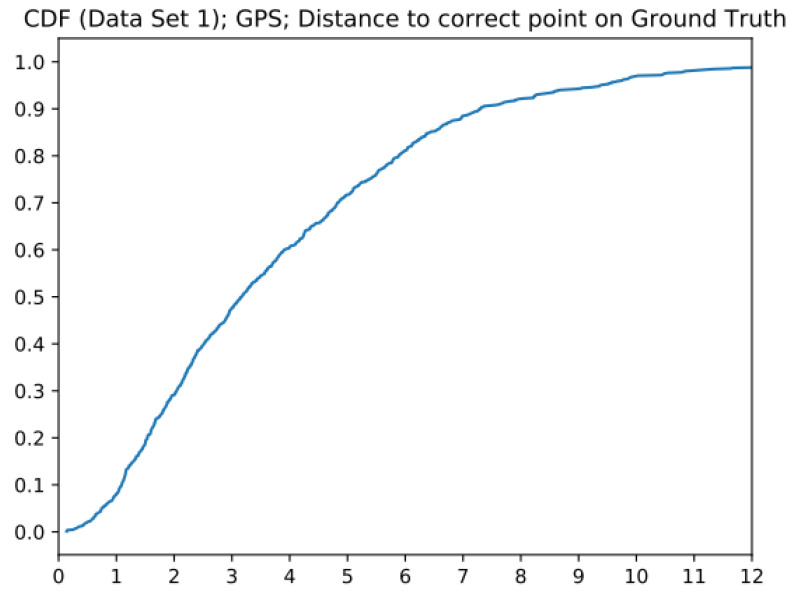
GPS error CDF of distance to correct point on the ground truth.

To counter the just-mentioned downside of only taking the distance to ground truth, this method measures the distance of a recorded location to the correct point on the ground truth. Because the datasets were recorded walking at a steady pace, it can be derived that the user is at 50% of the path after 50% of the elapsed time. It can be seen that the MSEs (Table 2) of all Kalman filters were significantly lower than from GPS alone. The downside of this method is that it is more dependent on the recording of a walk and the pace of the walk. The cumulative distribution function of the error is plotted in Figure 14.

#### 6.1.5. Testing Method 3: Distance Start to End

This method calculates the distance between the start and the endpoint. This testing is useful when used on Dataset 1 since those walks end at the same location as they start. The resulting distance was then set in relation to the length of the path. Therefore, as an example, a path length of 80.63 m and a start-to-end distance of 67 cm results in a 0.83% error. Using this method for assessing the Kalman filters is less valuable. Kalman filters take bad estimates as the initial values and improve with every following input value. The filters try to estimate the user’s position in a global reference frame, not the exact movement. This was the case, as can be seen in Table 3.

#### 6.1.6. Testing Method 4: Length of Recorded Data

**Figure 15:**
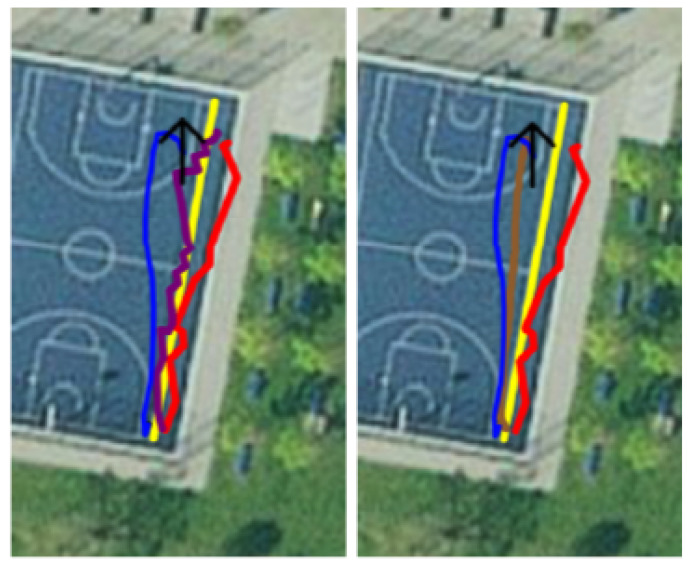
Jittering signal of KF Vel CT (**purple**) and smooth signal of KF CT (**brown**).

This method measures the length of the recorded data. It takes the distance between each location and accumulates it as the total length of the path. This method is valuable when looking at local reference frame systems and was used in the ARKit evaluation as well. It is also useful in testing KFs because a high error is an indication of a jitter in the data. An example of a jittering filter signal can be seen in Figure 15. The purple line shows the filter next to the GPS line (blue), the beacon line (red), and the smooth line (brown).

### 6.2. Raw Sensor Data

#### 6.2.1. GPS

**Figure 16:**
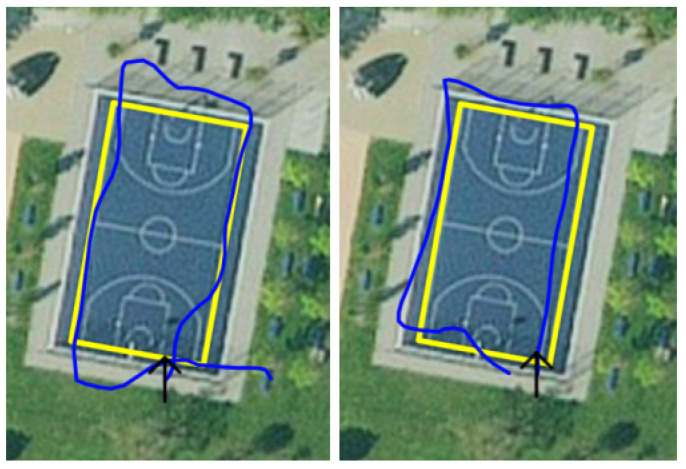
GPS signal in 15 November 2022 11:43:21.json (**left**) and 15 November 2022 11:34:23.json (**right**).

The evaluation produces values for all four testing methods. The most-precise one is the distance to the correct point on a path (Table 4). The mean error averaged over all 19 recordings was 3.69m, with an average standard deviation of 2m. The average distance between the start and end found in Table 3, which was 3.55m, showed about the same accuracy. In Table 4, it can be seen that there were maximal errors of up to 31.94m, and also, the median of each recording was lower than the mean. This is a sign of a generally good performance with rare outliers. These outliers seem to be often in connection to a bad GPS signal at the beginning of a recording, when there is not yet a good signal from more than a few satellites. An example of a somewhat bad and somewhat good GPS signal can be found in Figure 16. The left image shows a bad signal including an initial outlier in the right lower corner. The right image shows a clearly better signal.

#### 6.2.2. Bluetooth Beacons

For the evaluation of the Bluetooth beacon trilateration system, the Dataset 2 was used. By looking at Table 5, a mean error of 1.42 m and an MSE of 4.46 m averaged over all walks were seen. For the second testing method, the distance to the correct point on the ground truth (Table 6), the average values for the mean error of 3.72 m and the MSE of 19.10 m were about the same as for the GPS (mean: 3.52 m, MSE:19.89). The results of the third testing method with length-related data can be found in Table 7. The average length deviation of all walks was only +3%, but had a standard deviation of 24.74%. Even though the average was better than, for example, the GPS on this dataset, the very high standard deviation means the data were very inconsistent.

#### 6.2.3. Augmented Reality

For the purposes of display, the global position of the local coordinate system was associated with the first provided global position (GPS or beacons). Since the AR data are only given in a local reference frame, it did not make sense to evaluate them based on Testing Method 1 nor Testing Method 2. The evaluation was performed with the length and the distance start-to-end method. Table 8 shows an average deviation of a recorded length of −2.52% measured on both datasets. Table 9 shows an average of only a 0.83% distance error in relation to the path length. The standard deviation was also only 0.93%. In conclusion, it can be said that ARKit handles rotations in movement better than the actual distances of the movement itself.

### 6.3. Fusion: GPS and Augmented Reality

The evaluation of the fusion of GPS and augmented reality focused on Dataset 1. It can be seen from Table 1 that all four Kalman filters evaluated at about half the MSE of GPS or augmented reality. KF (mean: 1.23 m, σ 1.01 m, MSE 3.12 m) stood out with the best values. From Table 2, similar results can be seen. All four Kalman filters were better than GPS and AR alone. The outcome of the third testing method can be found in Table 10. As seen earlier, AR was very precise in length measurements, but measured on average −5.93% to little. Two filters managed to yield slightly better results in this category (KF CT: −4.87%; KF Vel CT: +5.59%). The bad performance of the other two filters might be traced back to jitter. It is unclear which of the four Kalman filters is the best to use.

### 6.4. Fusion Beacons and Augmented Reality

To assess the fusion of all three input signals, Dataset 2 was consolidated. As earlier, the good performance of the beacon system can be seen (Table 11). The values of all Kalman filters became worse than the values for fusion of just beacons and AR. In comparison to GPS, the filters performed still better, with KF Vel CT producing an 84% better result than GPS alone. The results of the second method are found in Table 12. This time, the addition of the GPS increased the performance. In the last section, using KF CT resulted in a 15% improvement over just using just beacons. When looking at the length testing method (Table 13), 3 of the 4 filters underperformed. KF, KF Vel, and KF Vel CT recorded all about 30% more length than the actual path length. This suggests high jitter in the data.

## 7. Discussion

The first observation that can be drawn is that the proposed beacon trilateration system yields better performance than GPS. The more complicated and conservative Method 2 returns only a 4% improvement in the MSE. However, the simpler Method 1, which was less precise, but also less prone to human recording errors, returned an 82% better MSE score for the Beacon system. With more extensive evaluation, mainly by increasing the walking distance and deploying more beacons, the two methods should converge.

### 7.1. Improvement over Raw Data

After evaluating the data, it can be concluded that all four Kalman filters improved the accuracy in user position over just using GPS or the proposed beacon trilateration system. Table 14 summarises the improvement of the best Kalman filter for each fusion category, split up into Method 1 and Method 2. The results from Method 1 suggest not taking GPS signals into account when a beacon system is available. Method 2 should theoretically result in a more accurate error measurement, but practically adds additional error caused by human imperfection.

### 7.2. Choice of Kalman Filter

When averaging over all testing methods and all fusion concepts, the proposed Kalman filter KF Vel CT reached the best average scores. Table 15 shows the average improvement in the MSE of KF Vel CT over the best-available raw input source.

### 7.3. Limitations and Future Work

#### 7.3.1. Kalman Filter

This work only considered Kalman filters as a data fusion method, which limits the system internally to linear models. Future projects should look at other algorithms of data fusion, such as the Extended Kalman Filter (EKS), the Unscented Kalman Filter (UKS), and the Particle Filter (PF). Those are capable of handling nonlinear models, which could increase the positioning accuracy. Furthermore, it makes the incorporation of the orientation into the main filter possible.

#### 7.3.2. Bluetooth Beacon Trilateration

There are some properties of the datasets that naturally limit the validity of the evaluation only to use cases that have the same properties. Both datasets were recorded outside, which results in full GPS coverage. The second dataset, which deployed beacons, is comparably small and does not have turns in its path. In future work, the evaluation should be extended by looking at more cases such as partial GPS coverage, indoor environments with deployed beacons, longer walking paths, and more independent smartphone motion including fast movements and different walking speeds.

#### 7.3.3. Navigation

The navigational information about the route that should be taken was solely based on the Apple MapKit framework as a routing service and inherited all the limitations that come with it. For example, even though the MapKit routes for pedestrians were used, MapKit does not have data about sidewalks. Therefore, the coordinates of the routes were all centred in the middle of the street. Furthermore, MapKit does not have indoor maps yet. In future work, maps that have more details could be used.

#### 7.3.4. Augmented Reality Visualisation

The occlusions by objects such as tree stems, traffic light poles, or other pedestrians were not handled at all. Alternative options regarding navigational clues could be used in the future, such as floating arrows or checkpoints that need to be traversed. Gathering more visual information about the environment is key when occlusions have to be properly handled.

## 8. Conclusions

This work introduced the idea of AR systems that know their global position and the possibilities this would open up for different location-based services. One example is navigation via AR based on precise global position and map-based routing data. AR navigation was approached with four different Kalman-filter-based algorithms that fused together information from ARKit, GPS, and a custom Bluetooth-based positioning system. The latter was needed, so that indoor environments with limited GPS availability are also supported. An extensive evaluation was performed in Section 6 with two main goals. The first was to determine which of the four Kalman filters performed best. This turned out to be the most complicated of the four, called KF Vel CT. The filter uses an internal state based on location and velocity and takes the ARKit data to control the inaccurate GPS and beacon locations. The evaluation’s second goal was to determine how much the accuracy could be improved when using data fusion. This turned out to be an interesting problem. It is not trivial to test how much more accurate than GPS a system is when GPS is the only available tool to determine global position. To address this, two main testing methods were proposed in combination with a satellite-image-based ground truth that was carefully constructed. An improvement in accuracy of 32% to 50% over raw signal data was shown. When AR, GPS, and the beacon system were fused, a median error of only 70cm was reached.

## Figures and Tables

**Table 1:** Evaluation (Dataset 1): GPS and AR; distance to ground truth.

	Mean	Median	max	min	σ	MSE
avg GPS	1.74	1.29	8.46	0.04	1.70	7.20
avg AR	2.04	1.78	5.37	0.00	1.29	7.78
avg KF	1.23	1.00	4.64	0.00	1.01	3.12
avg KF Vel	1.33	1.05	5.37	0.00	1.15	3.63
avg KF CT	1.26	1.03	4.28	0.03	0.96	3.13
avg KF Vel CT	1.43	1.08	4.62	0.03	1.18	3.81

**Table 2:** Evaluation (Dataset 1): GPS and AR; distance to correct point on the ground truth.

	Mean	Median	max	min	σ	MSE
avg GPS	3.80	3.54	9.70	0.77	2.03	22.57
avg AR	4.80	4.60	7.18	2.58	1.33	30.29
avg KF	3.63	3.21	6.82	1.13	1.55	19.27
avg KF Vel	3.58	3.27	6.86	0.91	1.57	18.60
avg KF CT	3.46	3.16	6.96	1.01	1.70	18.13
avg KF Vel CT	3.42	3.19	7.33	0.89	1.66	17.25

**Table 3:** Evaluation (Dataset 1): GPS and AR; distance between the start and end.

	Path Length	Distance Start to End	%
avg GPS	80.63	3.55	+4.40
avg AR	80.63	0.67	+0.83
avg KF	80.63	3.93	+4.88
avg KF Vel	80.63	3.75	+4.65
avg KF CT	80.63	4.02	+4.99
avg KF Vel CT	80.63	3.66	+4.54

**Table 4:** Evaluation (Datasets 1 and 2): GPS; distance to correct point on the ground truth.

	Mean	Median	max	min	σ	MSE
15 November 2022 11:31:16.json	5.48	5.10	13.07	0.14	3.02	39.14
15 November 2022 11:32:52.json	4.44	4.05	10.03	0.25	2.80	27.55
15 November 2022 11:34:23.json	2.59	2.32	5.95	0.60	1.37	8.59
15 November 2022 11:35:52.json	3.93	4.22	7.13	0.45	1.78	18.59
15 November 2022 11:43:21.json	7.52	7.25	11.34	4.23	1.91	60.16
15 November 2022 11:44:49.json	2.08	1.51	6.12	0.14	1.61	6.92
15 November 2022 11:46:08.json	2.28	2.19	4.28	0.72	0.89	6.00
15 November 2022 11:48:33.json	1.96	1.59	4.10	0.15	1.11	5.06
15 November 2022 11:49:46.json	2.77	2.78	4.23	0.81	0.85	8.37
15 November 2022 11:54:54.json	4.74	3.72	31.94	0.43	4.94	46.90
15 November 2022 11:56:08.json	4.07	4.17	8.49	0.55	2.11	20.98
15 November 2022 14:35:08.json	1.80	1.97	3.35	0.30	0.98	4.19
15 November 2022 14:41:31.json	2.68	2.76	5.92	1.05	1.08	8.37
15 November 2022 14:50:04.json	2.81	2.70	4.68	1.76	0.75	8.48
15 November 2022 14:52:00.json	5.16	3.64	11.58	0.56	3.59	39.49
15 November 2022 14:52:53.json	6.56	5.77	13.85	1.17	4.34	61.79
15 November 2022 14:53:46.json	2.92	3.22	3.93	0.72	1.02	9.59
15 November 2022 14:54:34.json	2.92	1.93	6.40	1.11	1.84	11.91
15 November 2022 14:55:25.json	3.32	2.82	7.33	0.69	2.06	15.29
average	3.69	3.35	8.62	0.83	2.00	21.44
σ	1.56	1.46	6.33	0.90	1.19	18.38

**Table 5:** Evaluation (Dataset 2): beacon; distance to the ground truth.

	Mean	Median	max	min	σ	MSE
15 November 2022 14:35:08.json	2.65	1.59	14.16	0.23	3.55	19.59
15 November 2022 14:41:31.json	1.44	1.20	3.46	0.11	1.04	3.16
15 November 2022 14:50:04.json	0.78	0.63	1.78	0.11	0.50	0.86
15 November 2022 14:52:00.json	1.20	1.14	2.66	0.13	0.69	1.91
15 November 2022 14:52:53.json	0.99	1.17	1.57	0.00	0.47	1.21
15 November 2022 14:53:46.json	1.15	1.07	3.54	0.05	0.78	1.91
15 November 2022 14:54:34.json	1.94	1.68	4.25	0.51	1.04	4.86
15 November 2022 14:55:25.json	1.21	1.30	3.20	0.09	0.84	2.16
average	1.42	1.22	4.33	0.16	1.11	4.46
σ	0.56	0.30	3.81	0.15	0.94	5.84

**Table 6:** Evaluation (Dataset 2): beacon; distance to correct point on the ground truth.

	Mean	Median	max	min	σ	MSE
15 November 2022 14:35:08.json	3.56	3.15	11.34	0.33	2.44	18.61
15 November 2022 14:41:31.json	3.56	3.55	6.41	0.33	1.64	15.34
15 November 2022 14:50:04.json	2.82	2.52	4.69	0.99	1.04	9.06
15 November 2022 14:52:00.json	4.97	4.23	10.20	0.78	3.62	37.78
15 November 2022 14:52:53.json	4.51	2.58	11.10	0.83	3.59	33.21
15 November 2022 14:53:46.json	3.18	2.89	4.99	1.81	1.08	11.26
15 November 2022 14:54:34.json	3.04	2.75	4.68	1.93	0.85	9.95
15 November 2022 14:55:25.json	4.11	4.45	5.14	2.07	0.84	17.55
average	3.72	3.27	7.32	1.13	1.89	19.10
σ	0.70	0.69	2.82	0.66	1.11	10.07

**Table 7:** Evaluation (Dataset 2): beacon; length-related data.

	Path Length	Recorded Length	Deviation (%)
15 November 2022 14:35:08.json	25.90	75.88	+65.86
15 November 2022 14:41:31.json	25.90	25.54	−1.42
15 November 2022 14:50:04.json	25.90	23.45	−10.47
15 November 2022 14:52:00.json	25.90	24.13	−7.35
15 November 2022 14:52:53.json	25.90	21.93	−18.12
15 November 2022 14:53:46.json	25.90	23.64	−9.56
15 November 2022 14:54:34.json	25.90	25.50	−1.60
15 November 2022 14:55:25.json	25.90	27.75	+6.66
average	25.90	30.98	+3.00
σ	0.00	17.05	24.74

**Table 8:** Evaluation (Dataset 1 and 2): AR; length-related data.

	Path Length	Recorded Length	Deviation (%)
15 November 2022 11:31:16.json	80.63	73.38	−9.88
15 November 2022 11:32:52.json	80.63	76.29	−5.69
15 November 2022 11:34:23.json	80.63	75.47	−6.84
15 November 2022 11:35:52.json	80.63	75.15	−7.30
15 November 2022 11:43:21.json	80.63	80.33	−0.38
15 November 2022 11:44:49.json	80.63	76.12	−5.93
15 November 2022 11:46:08.json	80.63	75.47	−6.85
15 November 2022 11:48:33.json	80.63	77.15	−4.52
15 November 2022 11:49:46.json	80.63	75.19	−7.24
15 November 2022 11:54:54.json	80.63	76.87	−4.90
15 November 2022 11:56:08.json	80.63	76.29	−5.69
15 November 2022 14:35:08.json	25.90	27.76	+6.70
15 November 2022 14:41:31.json	25.90	24.22	−6.96
15 November 2022 14:50:04.json	25.90	30.67	+15.55
15 November 2022 14:52:00.json	25.90	24.86	−4.20
15 November 2022 14:52:53.json	25.90	25.48	−1.65
15 November 2022 14:53:46.json	25.90	25.15	−2.98
15 November 2022 14:54:34.json	25.90	30.52	+15.14
15 November 2022 14:55:25.json	25.90	24.85	−4.22
average	57.59	55.33	−2.52
σ	27.02	24.51	7.01

**Table 9:** Evaluation (Dataset 1): AR; distance between start and end.

	Path Length	Distance Start to End	%
25 July 2022 11:31:16.json	80.63	1.34	+1.66
25 July 2022 11:32:52.json	80.63	0.52	+0.65
25 July 2022 11:34:23.json	80.63	0.17	+0.21
25 July 2022 11:35:52.json	80.63	0.13	+0.16
15 November 2022 11:43:21.json	80.63	2.74	+3.39
15 November 2022 11:44:49.json	80.63	0.13	+0.16
15 November 2022 11:46:08.json	80.63	0.29	+0.35
15 November 2022 11:48:33.json	80.63	0.05	+0.06
15 November 2022 11:49:46.json	80.63	0.67	+0.84
15 November 2022 11:54:54.json	80.63	0.46	+0.57
15 November 2022 11:56:08.json	80.63	0.85	+1.06
average	80.63	0.67	+0.83
σ	0.00	0.75	0.93

**Table 10:** Evaluation (Dataset 1): GPS and AR; length-related data.

	Path Length	Recorded Length	Deviation (%)
avg GPS	80.63	99.93	+14.01
avg AR	80.63	76.16	−5.93
avg KF	80.63	89.06	+9.18
avg KF Vel	80.63	93.04	+12.71
avg KF CT	80.63	77.14	−4.87
avg KF Vel CT	80.63	86.09	+5.59

**Table 11:** Evaluation (Dataset 2): GPS, beacon, and AR; distance to the ground truth.

	Mean	Median	max	min	σ	MSE
avg GPS	3.07	1.83	9.97	0.28	2.78	24.43
avg Beacon	1.42	1.22	4.33	0.16	1.11	4.46
avg AR	3.50	3.15	8.58	1.18	2.08	34.07
avg KF	1.70	1.16	5.38	0.16	1.39	8.00
avg KF Vel	1.89	1.12	6.54	0.07	1.66	11.32
avg KF CT	2.64	1.92	6.73	0.48	1.80	22.43
avg KF Vel CT	1.17	0.70	5.13	0.02	1.25	3.92

**Table 12:** Evaluation (Dataset 2): GPS, beacon, and AR; distance to correct point on the ground truth.

	Mean	Median	max	min	σ	MSE
avg GPS	3.52	3.10	7.13	0.92	1.96	19.89
avg Beacon	3.72	3.27	7.32	1.13	1.89	19.10
avg AR	4.33	4.24	6.90	1.79	1.45	24.65
avg KF	2.93	2.61	5.83	0.44	1.70	14.08
avg KF Vel	2.96	2.71	5.99	0.57	1.70	14.29
avg KF CT	2.80	2.51	5.48	0.72	1.52	13.00
avg KF Vel CT	2.71	2.22	6.26	0.36	1.77	13.76

**Table 13:** Evaluation (Data Set 2); GPS and Beacon and AR; Length related data.

	Path Length	Recorded Length	Deviation (%)
avg GPS	25.90	28.80	+9.48
avg Beacon	25.90	30.98	+3.00
avg AR	25.90	26.69	+2.17
avg KF	25.90	36.34	+28.54
avg KF Vel	25.90	38.67	+32.48
avg KF CT	25.90	25.99	+0.01
avg KF Vel CT	25.90	38.97	+31.46

**Table 14:** MSE improvement of the best Kalman filters.

	GPS and AR	Beacon and AR	GPS, Beacon, and AR
Method 1	+57%; KF	+50%; KF Vel CT	+12% to beacons; KF Vel CT
Method 2	+24%; KF Vel CT	+15%; KF CT	+32% to beacons; KF Vel CT

**Table 15:** MSE improvement of KF Vel CT.

	GPS and AR	Beacon and AR	GPS, Beacon, and AR
Method 1	+51%; KF	+50%; KF Vel CT	+12% to beacons; KF Vel CT
Method 2	+24%; KF Vel CT	+13%; KF CT	+32% to beacons; KF Vel CT

## Data Availability

Not applicable.

## Abbreviations

The following abbreviations are used in this manuscript:

AR	Augmented Reality
BLE	Bluetooth Low-Energy
EKS	Extended Kalman Filter
GNSS	Global Navigation Satellite System
GPS	Global Positioning System
HMD	Head-Mounted Displays
IMU	Inertial Measurement Unit
IoT	Internet of Things
JSON	JavaScript Object Notation
KF	Kalman Filter
KS	Kalman Smoother
MSE	Mean-Squared Error
PAReNt	Pedestrian Augmented Reality
PDA	Personal Digital Assistant
PDR	Pedestrian Dead Reckoning
RSSI	Received Signal Strength Indicator
SDK	Software Development Kit
SIMD	Single-Instruction Multiple-Data
UKS	Unscented Kalman Filter
UUID	Universally Unique IDentifier
